# Real-time data analysis for medical diagnosis using FPGA-accelerated neural networks

**DOI:** 10.1186/s12859-018-2505-7

**Published:** 2018-12-21

**Authors:** Ahmed Sanaullah, Chen Yang, Yuri Alexeev, Kazutomo Yoshii, Martin C. Herbordt

**Affiliations:** 1 Computer Architecture and Automated Design Lab, Boston University, Boston, MA, USA; 2 Argonne Leadership Computing Facility, Argonne National Laboratory, Lemont, IL, USA; 3 Mathematics and Computer Science Division, Argonne National Laboratory, Lemont, IL USA

**Keywords:** FPGA, Machine learning, Multi-layer perceptrons, Real-time, Inference, Cancer, Mass-spectrometry

## Abstract

**Background:**

Real-time analysis of patient data during medical procedures can provide vital diagnostic feedback that significantly improves chances of success. With sensors becoming increasingly fast, frameworks such as Deep Neural Networks are required to perform calculations within the strict timing constraints for real-time operation. However, traditional computing platforms responsible for running these algorithms incur a large overhead due to communication protocols, memory accesses, and static (often generic) architectures. In this work, we implement a low-latency Multi-Layer Perceptron (MLP) processor using Field Programmable Gate Arrays (FPGAs). Unlike CPUs and Graphics Processing Units (GPUs), our FPGA-based design can directly interface sensors, storage devices, display devices and even actuators, thus reducing the delays of data movement between ports and compute pipelines. Moreover, the compute pipelines themselves are tailored specifically to the application, improving resource utilization and reducing idle cycles. We demonstrate the effectiveness of our approach using mass-spectrometry data sets for real-time cancer detection.

**Results:**

We demonstrate that correct parameter sizing, based on the application, can reduce latency by 20% on average. Furthermore, we show that in an application with tightly coupled data-path and latency constraints, having a large amount of computing resources can actually reduce performance. Using mass-spectrometry benchmarks, we show that our proposed FPGA design outperforms both CPU and GPU implementations, with an average speedup of 144x and 21x, respectively.

**Conclusion:**

In our work, we demonstrate the importance of application-specific optimizations in order to minimize latency and maximize resource utilization for MLP inference. By directly interfacing and processing sensor data with ultra-low latency, FPGAs can perform real-time analysis during procedures and provide diagnostic feedback that can be critical to achieving higher percentages of successful patient outcomes.

## Background

Machine learning (ML) plays an integral part in solving many key scientific problems. It has been applied to such varied domains as finding cancer treatments [1], weather simulations [2], and design of new nanocatalysts [3]. Machine learning methods have been used in medical applications for many years [4]. First of all, machine learning can be used in monitoring patients’ vital signs. Muller, et al. introduced a Brain-Computer Interface (BCI) [5] based technique that can extract appropriate features from continuous EEG signal [6]. The proposed system collects a number of trials from patients while they are asked to perform fixed tasks. Using the collected datasets as training data, the system infers the typical EEG patterns in real-time. In [7], Shoeb and Guttag presented a machine learning algorithm to recognize epileptic seizure from the scaled EEG signal. Machine learning is also widely used in medical image retrieval, enhancement, processing and mapping as introduced in [8–12]. Zacharaki, et al. performed a study on distinguishing different types of brain tumors based on Support Vector Machine Recursive Feature Elimination (SVM-RFE) algorithm [13]. Pereira et al. introduced an approach to use machine learning algorithms to decode variables of interest from fMRI data [14]. Apart from that, machine learning is also an effective supplement in diagnosing diseases [15, 16]. Ozcift, et al. constructed Rotation Forest (RF) ensemble classifiers for multiple machine learning algorithms in a computer-aided diagnosis (CADx) system that is used for diagnosing diseases like diabetes and Parkinson [17]. Li and Zhou proposed a semi-supervised learning algorithm, Co-Forest, that uses undiagnosed samples along with only a small amount of diagnosed ones as training datasets for CAD systems targeting breast cancer diagnosis [18].

Multi-Layer Perceptrons (MLPs) are an important subset of ML that not only optimize existing applications and practices but also widen that pool by enabling designs to meet more stringent requirements of reliability, performance, complexity, and portability that are not possible from traditional approaches. MLPs consist of multiple layers of firing neurons, with each layer using responses of previous neurons as stimulus. Use of MLPs is divided into two stages: training and inference. Training is an iterative process that determines neuron connection strengths in a given MLP. We assign initial values to connection strengths and then apply training cases as inputs to the model. The correct results of training cases are known beforehand, which we refer to as expected values. Corresponding measured outputs of the model are compared with these expected values, and connection strengths are then updated by a (static or dynamic) factor in order to minimize the error between the two sets of results. This process is repeated until measured results converge to values that reduce errors to within acceptable bounds. Training is typically an offline operation and thus does not impact analysis timeframes. Inference, on the other hand, is performed in real-time and refers to the process of performing classification (or regression) on a set of test input cases using the trained model. In our work, we focus on improving inference latency in order to achieve small analysis timeframes.

Traditional processors running inference algorithms cannot meet the required timing constraints in most cases due to the large overhead of communication protocols, memory accesses, and static (often generic) architectures. Data must first be moved from sensors to CPU buffers, typically by using serial ports, thus limiting the bandwidth. For CPU-based implementations, processing individual test cases results in a large number of cache misses. This is because MLPs have virtually no data reuse for the same test case, and batch processing is not possible due to latency bounds. Moreover, meaningful model sizes tend to be larger than the higher cache levels (closer to the CPU) and hence entire models cannot fit in the L1 or L2 cache. For GPU implementations, further memory transactions are required to move data to and from the device memory over the PCIe bus, which increases the overhead. Low data reuse and batch-less processing also hurt GPU performance since the computations may not be sufficiently parallel to fully utilize the thousands of available cores. For cores that are assigned work, a significant number of cycles are likely to be idle as threads wait for off-chip data to be accessed. ASIC based designs have typically managed to fill this gap by providing massive amounts of resources and specialized pipelines that are tailored for Deep Neural Networks (DNNs); one example is the Google TPU [19]. However, as the number of diverse applications and their associated models grows, these ASICs effectively address a domain, rather than a particular application, and hence are unable utilize application-specific optimizations at the level needed.

Reconfigurable architectures, such as FPGAs, are becoming increasingly popular since their logic can be configured to construct application-specific architectures [20–25]. For MLPs in particular, arbitrary sized models can be easily implemented, scaled, and even transferred to newer generation technologies by recompiling the design. Like GPUs, FPGAs are Commercial-Off-The-Shelf (COTS), which means users can buy the device, generate logic for their desired model, and ensure that the resulting compute pipelines are optimal not only for MLPs, but also for their specific MLP application. Moreover, since the underlying computation structure does not change, complex HDL codes do not have to be written. Instead, simple scripts can be used to create the required architecture. As FPGA on-chip memory grows, reaching several megabytes in the current generation, users can move all model parameters to the on-chip SRAM and remove idle cycles caused by fetching weights from off-chip DRAM. Moreover, FPGAs offer support for most common serial and parallel protocols and can thus further minimize the latency of memory and I/O transactions by supplying data directly (from sensors) to compute pipelines. All these features result in a transition of the computation from memory bound to compute bound.

Improving latency (and hence performance) of compute-bound MLP inference requires all pipelines to operate both stall free and at high bandwidth. The former is achieved by ensuring modules in the design source/sink data at rates that are constrained by the latter. Since modules in MLP architectures are tightly coupled and operate within the same clock domain, constraints can occur even for indirect connectivity. Therefore, by selecting higher interface bandwidths for particular ports, the complexity (and hence latency) of potentially multiple modules can become significantly large. This increase in complexity can outweigh the benefits of higher module throughput and thus increase the overall latency of the computation.

FPGA-based MLP implementations have received significantly less attention than other DNNs such as Convolutional Neural Networks. The most prominent design is Microsoft’s FPGA-based inference architecture, Brainwave [26], targeting low compute-to-data ratio DNN applications such as MLPs, Long Short-Term Memories (LSTMs), and Gated Recurrent Units (GRUs). The memory bandwidth bound is alleviated by using on-chip block RAM for weight matrix storage. For an 8-bit integer implementation on Stratix V FPGAs, Brainwave achieves 2.0 TOps/s, while on Stratix 10 FPGAs, they claim to have 31 TOps/s performance running at 500 MHz. In [27, 28], the authors proposed FPGA-based MLP architectures, but their work serves as a proof-of-concept and is also constrained by off-chip memory bandwidth. Sharma et al. presented an automated DNN design generation framework, DNNWeaver [29], which also depends on DRAM access speed for performance. Moreover, the DNNWeaver compute units are constrained to Digital Signal Processor (DSP)-only implementations and logic cells are not used for ALUs. Gomperts et al. introduce a general purpose architecture for MLP based on FPGAs [30]. Their design generates individual processing elements for each layer, which is not feasible for large neural networks where resources on a single FPGA may not even be sufficient to compute a single layer in parallel.

With regard to stand-alone operation with a direct interface to sensors, FPGAs have shown support for various forms of connectivity without host support. Apart from General Purpose I/O pins [31, 32] that can implement virtually any communication protocol, they also offer direct chip-chip connectivity through Multi-Gigabit Transceivers (MGTs) and network connectivity through dedicated Ethernet controllers. MGTs are serial links that provide low-latency, high-bandwidth, and low-energy interfaces [33–38] and enable communication at rates of 100 Gbps per MGT. Current high-end FPGAs can have close to 100 MGTs. These MGTs can be used to connect multiple FPGAs together and perform computations for models that are too large for a single device. Large multi-FPGA clusters have long been a staple of computational finance and certain other industries. George et al. [39] presented an academic example of a 64 FPGA cluster with direct FPGA-to-FPGA links in a 3D torus network. Similarly, Microsoft’s original Catapult System [40, 41] consists of 1632 nodes with FPGAs directly interconnected via MGTs in a series of 6×8 tori.

In our work, we explore the application-aware optimization space for compute-bound MLP inference processors using our proposed FPGA-based architecture. By identifying modules in the critical path and their interconnectivity, we determine and optimize parameters for a given application in order to minimize the latency of the entire model evaluation and meet real-time constraints. This strategy enables our design to be feasible for applications such as real-time medical diagnosis.

## Methods

### Aim

In this study, we have designed and implemented a real-time Multi-Layer Perceptron inference processor for medical diagnosis using FPGAs. Our focus is on achieving ultra-low analysis latency to meet real-time constraints. The proposed system consists of a modular approach with standardized interfaces that enables hardware of individual functions to be easily modified based on changes to the inference model, design practices, or resource availability. We demonstrate that the ability to be application specific enables FPGA-based designs to choose architecture parameters that minimize latency and maximize utilization of computing resources. Using mass spectrometry benchmarks for cancer detection, we show that FPGAs can outperform traditional computing technologies such as CPUs and GPUs for real-time MLP applications.

### Hardware specifications

We have tested our designs on the Intel Arria 10 FPGA (AX115H3F34E2SGE3), which has 427,200 Adaptive Logic Modules (ALMs), 1518 DSP blocks, 53 megabits of on-chip memory, and a maximum of 624 user General Purpose Inputs/Outputs (GPIOs) [42]. The GPU used is a Tesla P100 which has 3594 CUDA cores and a 12 gigabyte High Bandwidth Memory (HBM2) with a peak bandwidth of 549 GB/s. For the baseline, we use an eight-core 2.6 GHz Intel Xeon E5-2650v2 CPU.

### Software specifications

FPGA designs are implemented by using Altera OpenCL 16.0.2 [43] to ensure that standard optimizations are applied and the impact of varying design parameters on latency can be fairly determined. Resource usage is measured by using the Quartus Prime Pro 16.0 compiler. GPU reference designs are compiled by using TensorFlow [44] r1.4, python 3.6.2, cuDNN 6.0 and CUDA 8.0. CPU code is also compiled by using TensorFlow r1.4. Both GPU and CPU designs use single-precision floating point, while the FPGA implementation uses fixed-point arithmetic, with arbitrary variable sizes that are optimized for each computation stage.

### Benchmarks

We demonstrate the effectiveness of the FPGA-based MLP inference processor by evaluating models for detecting cancer using protein profiles. The two datasets used are obtained from [45] and use Surface-Enhanced Laser Desorption and Ionization (SELDI) protein mass spectrometry to generate data for ion intensities at 15154 mass/charge values. The first is Ovarian-8-7-02 (Ovarian), which contains 91 normal and 162 cancer cases. The second is Prostate Cancer Studies (JNCI), which contains 69 cancer and 63 normal cases. Cancer and normal data for both benchmarks are further divided into training and test sets, as shown in Table 1. For both benchmarks, we propose a model with two hidden layers, with the output determining whether the patient is normal or has cancer. We train the proposed models in single-precision floating point using Tensorflow and find that prediction accuracy for both benchmarks is > 90%. For the FPGA implementation, we convert trained parameters to 8 bits for weights and 32 bits for biases. This conversion is achieved by scaling them, based on a given layer’s maximum and minimum values, in order to utilize the entire integer range.

**Table 1:** Proposed MLP models for mass spectrometry benchmarks

Model	Dimensions	Training cases	Test cases	Accuracy	F_1_ Score
Ovarian	**15154** × 512 × 512 ×**2**	160	92	98.9%	0.99
JNCI	**15154** × 64 × 512 ×**2**	100	32	90.6%	0.92

The boldface represent the sizes of input and output layers of the Multi Layer Perceptron

## Multi layer perceptrons

**Fig. 1:**
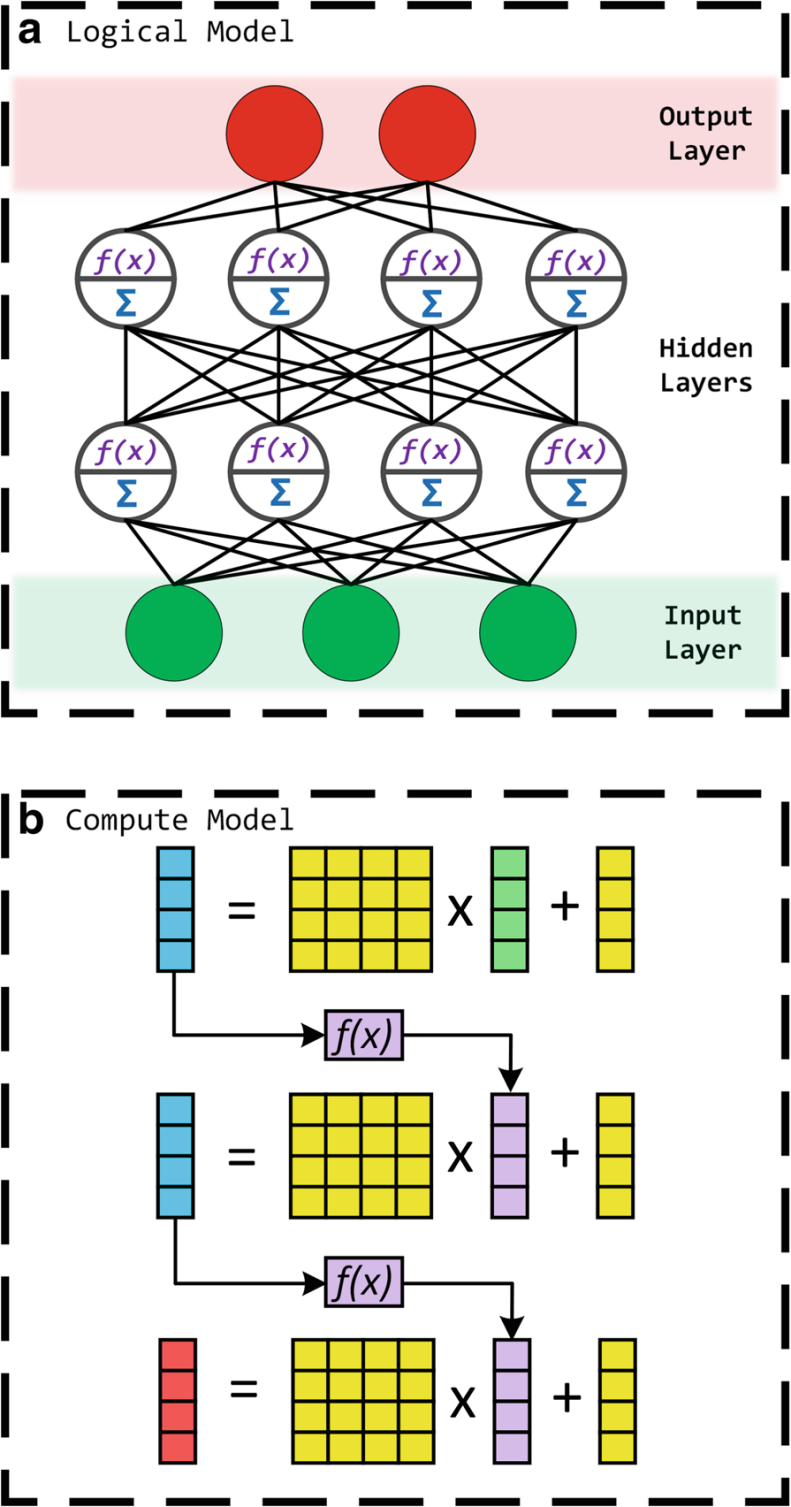
MLP models for (**a**) logical configuration and (**b**) compute. MLP models can have many layers, with each layer composed of potentially multiple neurons. Each neuron is connected to all neurons in the layer before it. The weights assigned to input connections of a single neuron are stored as a row vector, and every neuron has its own associated row vector. Combining row vectors of neurons in a layer forms the weight matrix for that particular layer. A non-linear function is applied to the weighted sum of a given neuron’s inputs, and the result forms the output of the neuron

In this section, we provide an overview of Multi-Layer Perceptron based neural networks using both logical and computational models. Multi-Layer Perceptron models are typically composed of an input layer containing feature values measured using sensors, an output layer containing the diagnosis result, and, potentially, multiple hidden layers that perform the required computations on the input data. Each layer consists of one or more neurons, depending on the model. MLPs are fully connected as illustrated in Fig. 1a: a neuron in any given layer is connected to the outputs of all neurons in the previous layer.

Inputs to the hidden and output layer neurons are scaled and accumulated by using weights (connection strengths) that are determined during training. A non-linear function, called the activation function, is applied to the result, which then becomes the neuron output. Figure 1b shows this operation represented as a Matrix Vector Multiplication (MVM). Each layer has a unique weight matrix, which contains connection strengths, and a bias vector. Layer inputs are the result vector from the previous layer, or the input vector if it is the first hidden layer. A given row from the *X*×*Y* weight matrix for a given layer represents the connection strengths of *Y* neurons in the previous layer assigned to one of the *X* neurons in the current layer. The activation function is applied to individual elements of the output vector. During the training process, all data are floating point. Classification can be performed by using integer arithmetic without loss of accuracy.

## MLP Inference architecture

**Fig. 2:**
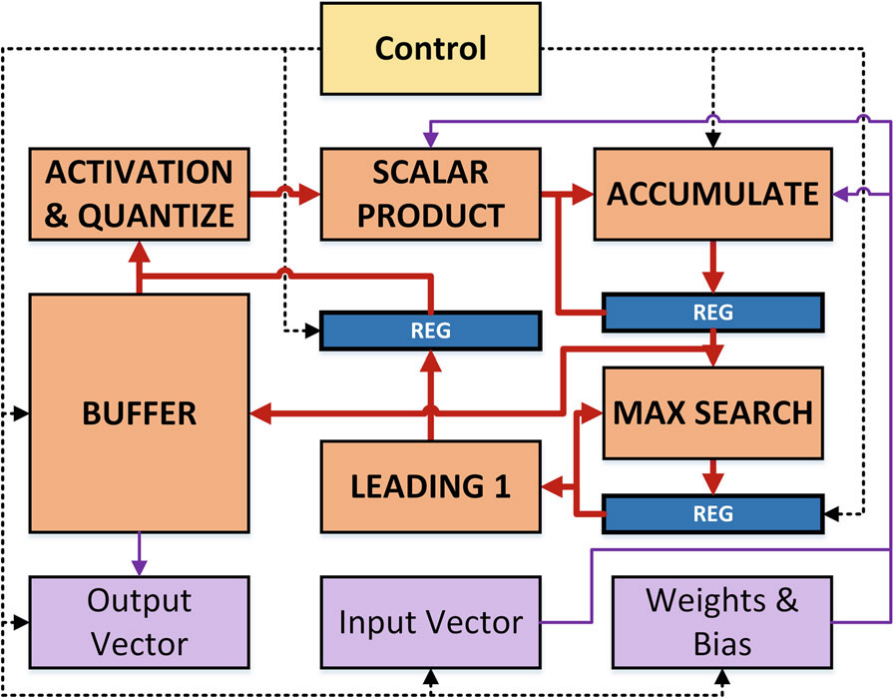
The architecture of the proposed low latency MLP inference system. The modular-centric approach and well-defined boundaries/interfaces enable updates, additions, and deletions to be performed easily and with minimum changes to adjacent components

In this section, we present the MLP inference architecture illustrated in Fig. 2. We use a modular approach to component design that enables parameters to be varied in order to implement the optimal dimensions for a given application. Compute and control planes are segregated, enabling additions, deletions, and updates to be easily performed. Individual components within each plane also have well-defined boundaries and interfaces to ensure that design changes can be performed at very high granularity, with minimal effort, and without necessitating changes to other logic beyond required parameter updates. Layers are processed sequentially, with modules performing all computations for a given layer before evaluating the next one.

### Compute

#### Scalar product

**Fig. 3:**
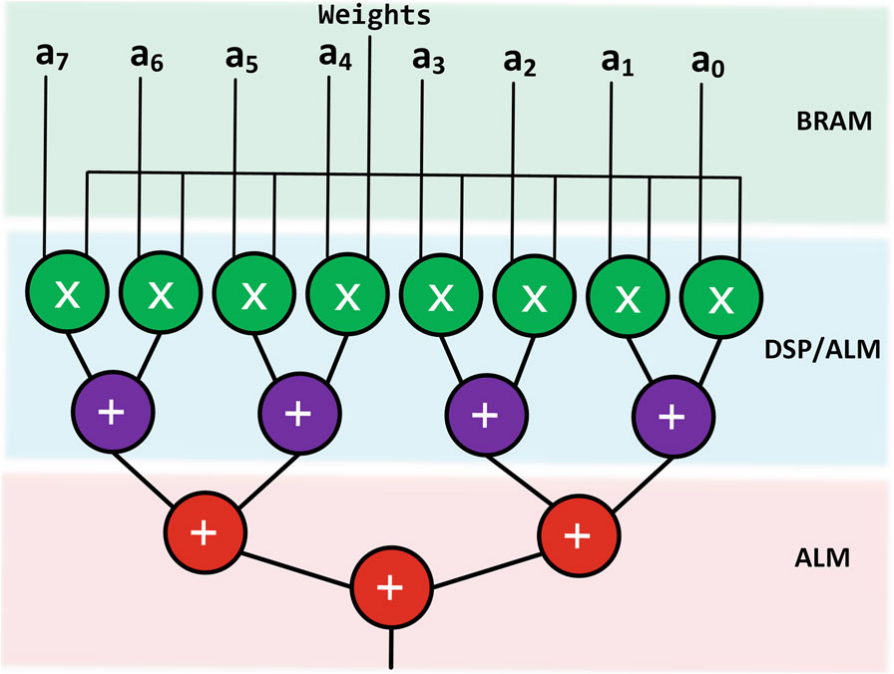
Scalar product module design using both DSP and ALM multipliers. Each scalar product slice is composed of DSP or ALM multipliers and an adder tree with logarithmic complexity to minimize latency. For DSP-based slices, two multipliers and one adder are implemented in DSP blocks while the remaining adder tree is always implemented by using ALMs

For this module, users can specify both the number of Scalar Product evaluations and their size. Computations are performed by using 8-bit variables, while results are accumulated into 32-bit outputs. To minimize latency, we use tree-based structures, rather than systolic arrays, for implementing the Scalar Product modules. This approach ensures that we can scale well to larger input vectors. Typical FPGA implementations focus primarily on DSP-based resources for this stage. However, we provide users with the capability of selecting arbitrary numbers of DSP and ALM-based multipliers at the granularity of a Multiply-Add module as shown in Fig. 3. Based on board resources, users can specify the number of available DSPs while the remaining computing entities are synthesized with ALMs.

#### Accumulate

If the size of a Scalar Product module is smaller than the input vector, multiple iterations are required to accumulate partial sums and obtain a final value. Moreover, a bias value must be added to the sum. The Accumulate module performs all of these functions by using dedicated *Accumulate Registers*. It receives triggers from the control plane on whether to accumulate or to re-initialize the registers for a new operation cycle.

#### Activation & requantization

The Activation & Quantization module reads data from the buffer, performs 32-bit ReLU activation (*RELU*(*x*)=*max*(*x*,0)), and then quantizes data back to 8 bits for the next layer. Quantization is performed by using truncation because of the high costs of division hardware. Because of the nature of the operation (compression), the difference in results is small in this particular context. Moreover, ReLU activation ensures that the Most Significant Bit (MSB) of our 8-bit result is always 0. Therefore the effective compression target is 7 bits, which further reduces the difference between division and truncation results.

#### Max search

Being able to perform quantization requires knowledge of the upper and lower data limits. Because of the ReLU activation, we are guaranteed a lower limit of 0. Searching for the upper limit must be done without stalling the data stream. Consequently, we use the Max Search module to perform local maxima searches on data as it becomes available and update an associated register if a local maximum exceeds the current global maximum. This approach ensures that latency is based on the dimensions of the accumulator outputs and not the full input vector. Employing a tree-based search further reduces the delay.

#### Leading 1

Once the maximum value for a given output has been determined, we use the Leading 1 module to find the most significant non-zero bit and use this position to perform truncations for quantization. The output is constrained to be between 6 and 30 (on a scale of 0-31) since the former means all values are already within 8 bits while the latter represents the largest possible positive numbers. As with Scalar Product and Max Search, the evaluation is performed with logarithmic complexity.

#### Buffer

**Fig. 4:**
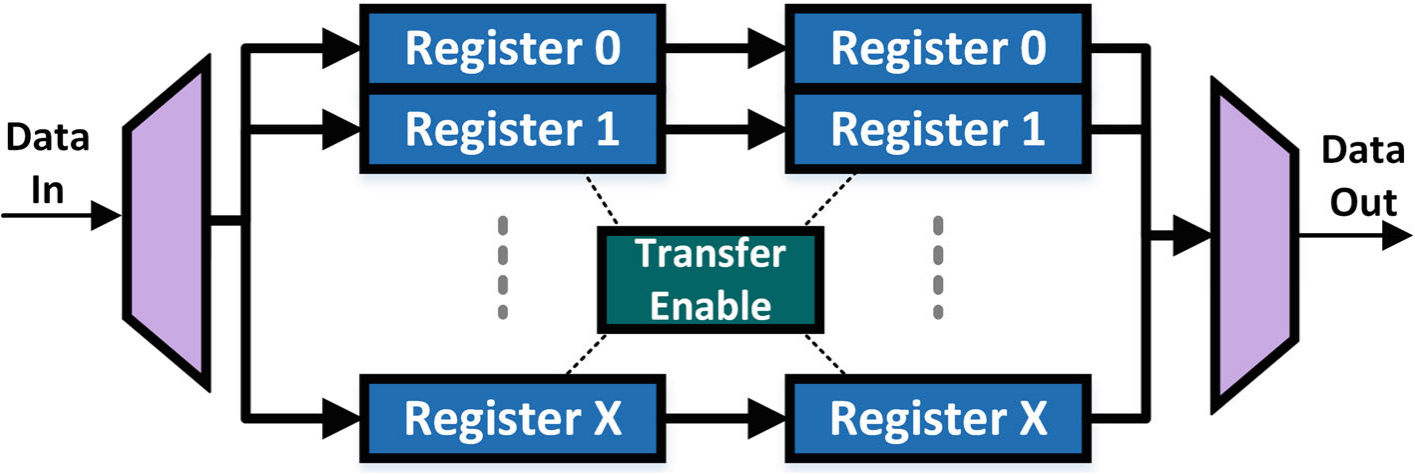
Structure of the Buffer Module. The two-stage design enables us to hold values of a previous layer (input vector to Scalar Product) while also storing results of current layer computations

The Buffer module stores result vectors for both the current and the previous layer (input to current layer). While the Buffer is used purely as a memory resource, it is included in our compute plane because of its tight coupling with the Accumulate and Activation & Quantize modules. It is implemented by using registers in order to meet throughput demands for architecture-specific source and sink sizes. A two-bank architecture, Fig. 4, comprising of separate input and output memory banks is used. The output memory bank stores results from the previous layer and supplies this data to the Activation & Requantization module. On the other hand, the input memory bank stores results of ongoing computations by sinking data from the Accumulate Registers. A single-cycle data transfer, from the input bank to the output one, is triggered once all neuron outputs have been computed and the system is processing the global maximum. This ensures that data are ready to be supplied when the next layer is picked up for processing.

#### Critical path

Of the compute plane modules discussed above, all but the Buffer lie in the critical path. We divide these modules into two categories; the Variable Critical Path (VCP) and the Persistent Critical Path (PCP). The Variable Critical Path is entirely the Scalar Product module. It is equal to the number of calls needed for the Scalar Product module to perform all multiplication operations. Since it is based on the relative dimensions of the weight matrix and Scalar Product module, it will vary for each layer. Once the last set of results has been produced, modules that need to evaluate this last result before a new layer can be processed are referred to as the Persistent Critical Path. It corresponds to a fixed number of cycles independent of layer dimensions. Applicable modules include Accumulator (with register), Max Search (with register), Leading 1, and Activation & Quantize.

#### Design parameters

Because of the tight coupling of our system design and the latency requirement, certain modules should be able to sink stall-free the entire throughput of their preceding component, as well as source the required throughput to their subsequent one. A chain of such modules where this throughput cannot be modified specifies a design parameter. In the proposed architecture, two such chains exist.

The first chain connects the Buffer and Scalar Product modules. It constrains i) the output size of the Buffer, ii) the size of the Activation & Quantize module, and iii) the length of a vector input to the Scalar Product module. We refer to this parameter as *M*. The value of M determines the number of columns being processed in the weight matrix. The second chain connects the Scalar Product to the Max Search module. It constrains i) the number of Scalar Product units, ii) the number of parallel accumulators needed to add partial sums, and iii) the number of elements over which to perform a maximum value search. We refer to this parameter as *N*. The value of N determines the number of rows being processed in the weight matrix. A third chain, between Max Search and Leading 1 modules, always has a throughput of one 32-bit element per cycle and hence does not have a variable design parameter. In our work, we explore the impact of varying M and N on performance.

#### Latency model

We present an average-latency model for the proposed architecture. We assume standard design practices for implementing the three predominant types of entities: multipliers, pipeline stages, and trees. Equation 1 gives a generic model for latency that can be used to describe combinations of the above. *A* is a multiplicative factor representing the number of pipeline stages per tree layer. *B* refers to the number of variables reduced by the tree while the logarithm computes its corresponding depth. *C* is a constant latency offset representing the minimum cycles taken by any module to perform its assigned computation. *D* represents multiple module calls and is applicable only to modules in the Variable Critical Path, i.e., the Scalar Product multiplication stage.

1 $$  {module}_{i} = {D}_{i}\left({A}_{i}\lceil{log}_{2}({B}_{i})\rceil + {C}_{i}\right)\;cycles  $$

Based on the general model above, we determine the total system latency for a given application. The motivation here is to determine the values for M and N that minimize the overall latency, rather than that of a particular layer. As shown in Eq. 2, the total latency is a linear combination of all latencies across all *K* layers of the application. We assume that the Accumulate and Activation & Quantize operations occur in a single cycle (as there are no data dependencies between vector elements and simple computation stages). Max Value Search and Leading 1 are both modeled as trees, but the latter one has constant latency due to a fixed input size (32 bits). We separate the Scalar Product module into multiplication and accumulation stages. The former is modeled as a single-stage pipeline that processes M ×N blocks of a weight matrix per cycle. On the other hand, the accumulation stage is absorbed into the Persistent Critical Path and has logarithmic latency based on M.

2 $$ {\begin{aligned}  {L}_{Total} = & \sum\limits_{i=1}^{K} \left\{ \left\lceil \frac{{rows}_{i}}{N}\right\rceil*\left\lceil\frac{{cols}_{i}}{M}\right\rceil + \lceil log_{2}(M)\rceil + (log_{2}(1)+1) \,+\, 1 \,+\, \lceil log_{2}(N)\rceil + 1 \right.\\ &\left. + log_{2}(32){\vphantom{\rceil*\lceil\frac{{cols}_{i}}{M}\rceil}}\right\} + \sum\limits_{i=1}^{K-1} \left\{log_{2}(1)+1\right\} \end{aligned}}  $$

By comparing the effective latencies of Variable (VCP) (Eq. 3) and Persistent (PCP) (Eq. 4) Critical Paths, we demonstrate the substantial impact of design parameters on performance. For larger values of M and N, the latency of VCP decreases, and fewer iterations are needed to perform all required multiplications for a given layer. However, this also causes the latency of PCP to increase and hence can potentially decrease performance despite there being more compute resources. On the other hand, having small values of M and N can still increase overall latency since a greater number of cycles are spent computing scalar products. These complex interactions highlight the need for reconfigurable architectures that can be tuned for a particular application.

3 $$  {L}_{VCP Total} = \sum\limits_{i=1}^{K} \lceil {{rows}_{i}}/{N}\rceil*\lceil{{cols}_{i}}/{M}\rceil  $$

4 $$  {L}_{PCP Total} = K(\lceil log(M)\rceil+\lceil log(N)\rceil+8)-1  $$

### Control

**Fig. 5:**
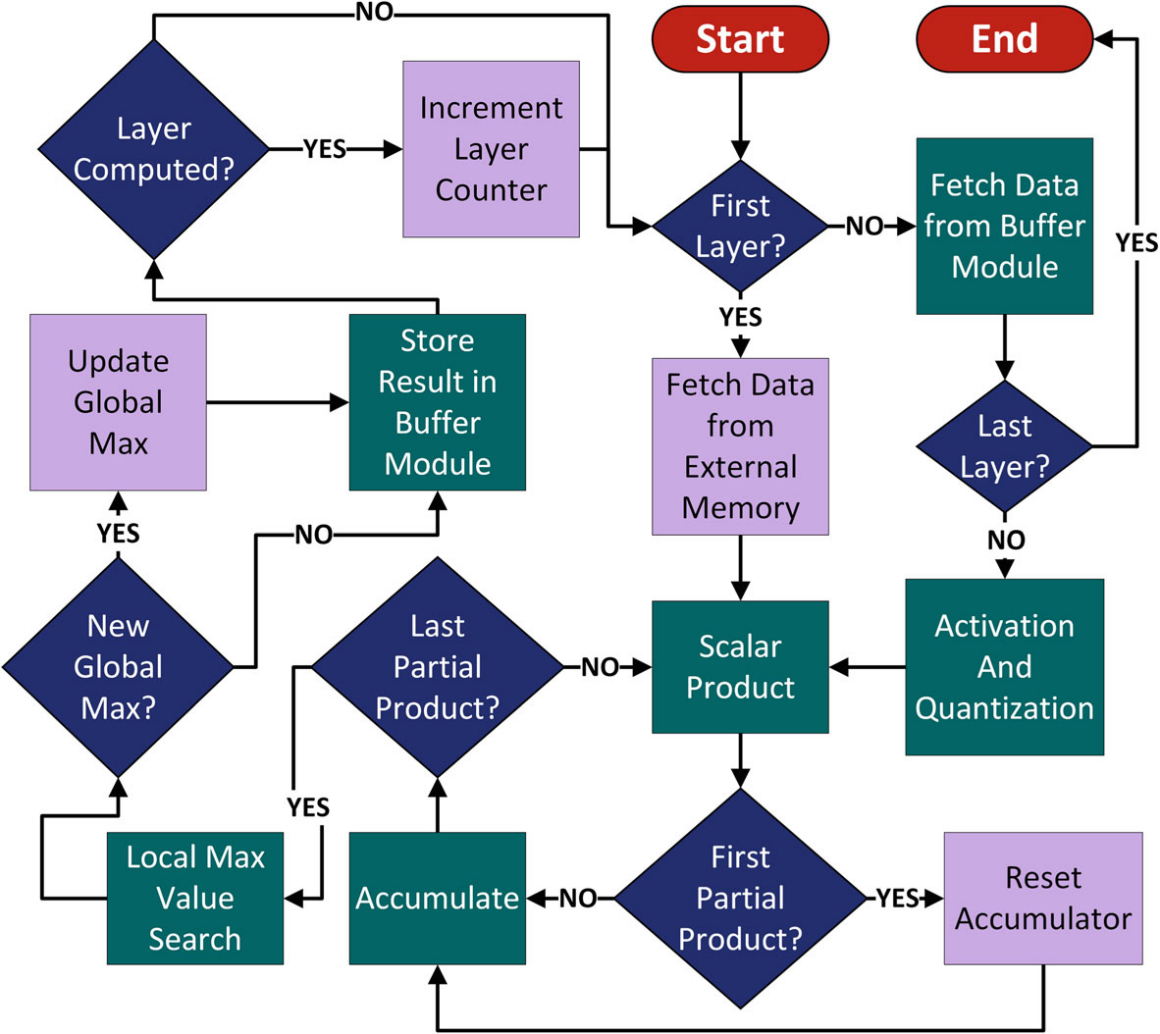
Algorithm: An overview of the algorithm used to determine the event triggers needed to control the flow of data in the inference processor

Using the modules and their functions outlined previously, Fig. 5 gives an overview of the algorithm for performing inference on a given test vector. The control unit is responsible for generating event triggers to coordinate the flow of data between different modules (green). These triggers are based on system states (blue) and include start/end indicators, variable updates (e.g., resets, increments, swap), read/write signals, and data source selection (e.g., the buffer module, external on-chip memory).

**Fig. 6:**
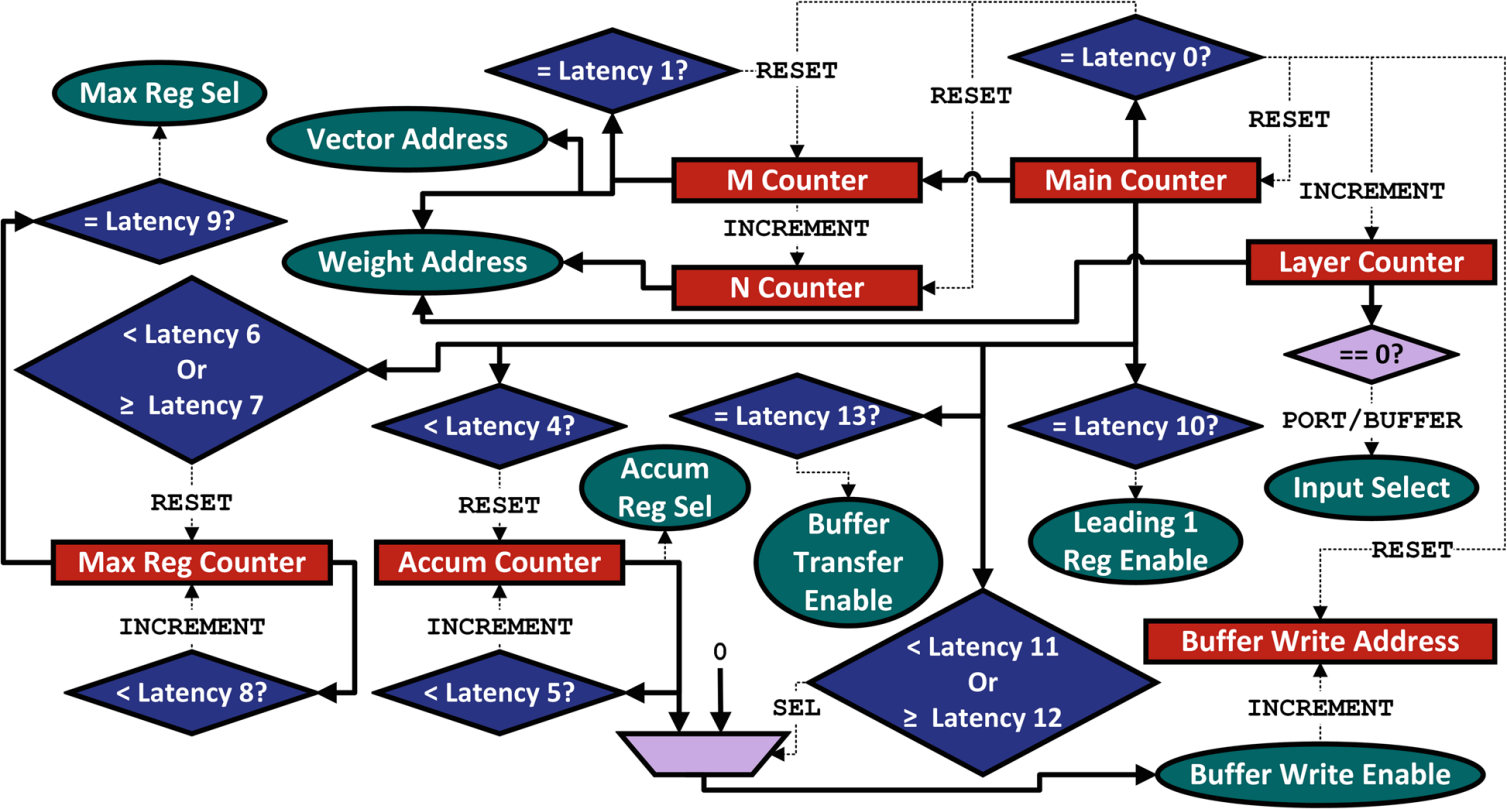
Control Unit: Execution of the entire application model can be done based on a single global counter without any feedback from modules or user-supplied run-time instructions. Values of the latency tags for each layer are hard-coded into the system and selected based on the layer counter value

A detailed implementation of the control unit is shown in Fig. 6. For given values of M and N and application model dimensions, we can determine how long each layer will take, at which cycle individual triggers will be given, and the computation being performed by each module at any given time. All these can be coordinated based on a single global counter (Main Counter). By having an instruction-free implementation, the overhead of fetching and decoding instructions is avoided, and end-to-end data flow for the entire application can be made stall free.

To define ranges and trigger points for setting and resetting values of control signals and state machine counters, we use latency tags. Each tag is based on the latency of an individual module in the corresponding data path. Table 2 lists these tags and their values. QA, MM, AC, MX, and LO refer to latencies of the Activation & Quantization, Scalar Product, Accumulate, Max Value Search, and Leading 1 modules, respectively. Constants represent latencies of registers at the output of the Accumulate and Max Value Search Modules. MBLOCKS and NBLOCKS refer to the number of blocks the weight matrix of a layer can be divided into in each dimension, while BLOCKS is the product of these, that is, the number of cycles needed for the entire weight matrix of a layer to be processed. Tags 2 and 3 are reserved for external connectivity in future work.

**Table 2:** Latency tags for defining trigger ranges

Tag	Value
Latency_0	L_QA(M) + L_MM(M,N) + BLOCKS[i] + L_AC(N) + 1 + L_MX(N) + 1 + L_LO
Latency_1	MBLOCKS[i]
Latency_4	L_QA(M) + L_MM(M,N)
Latency_5	MBLOCKS[i] -1
Latency_6	L_QA(M) + L_MM(M,N) + L_AC(N) + 1 + L_MX(N)
Latency_7	L_QA(M) + L_MM(M,N) + BLOCKS[i] + L_AC(N) + 1 + L_MX(N)
Latency_8	MBLOCKS-1
Latency_9	MBLOCKS-1
Latency_10	L_QA(M) + L_MM(M,N) + BLOCKS[i] + L_AC(N) + 1 + L_MX(N) + 1 + L_LO -1
Latency_11	L_QA(M) + L_MM(M,N) + L_AC(N) + 1
Latency_12	L_QA(M) + L_MM(M,N) + BLOCKS[i] + L_AC(N) + 1
Latency_13	L_QA(M) + L_MM(M,N) + BLOCKS[i] + L_AC(N) + 1

## Results

### Measured latency

**Fig. 7:**
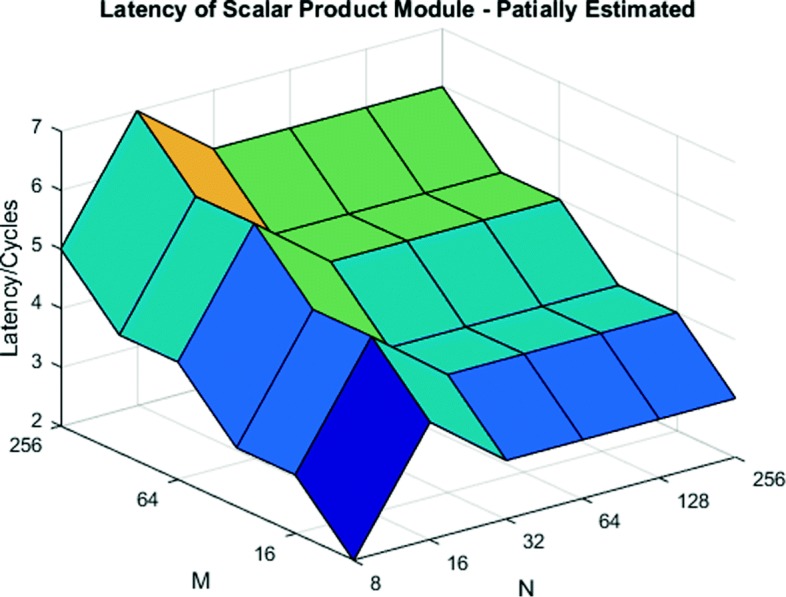
Latency comparison of various M and N for Scalar Product module. Latency increases with larger M (more tree stages) but is invariant in N (tree replication)

Figure 7 shows the latency of the Scalar Product module. Data points for (M ← 8:256, N ← 8), (M ← 8, N ← 8:256), (M ← 16, N ← 16), (M ← 32, N ← 32) and (M ← 64, N ← 64) are measured values while the remaining points are estimated based on observed trends. As illustrated by the graph, larger M values correspond to higher latency, while the latency due to N is mostly constant (due to simple design replication).

**Fig. 8:**
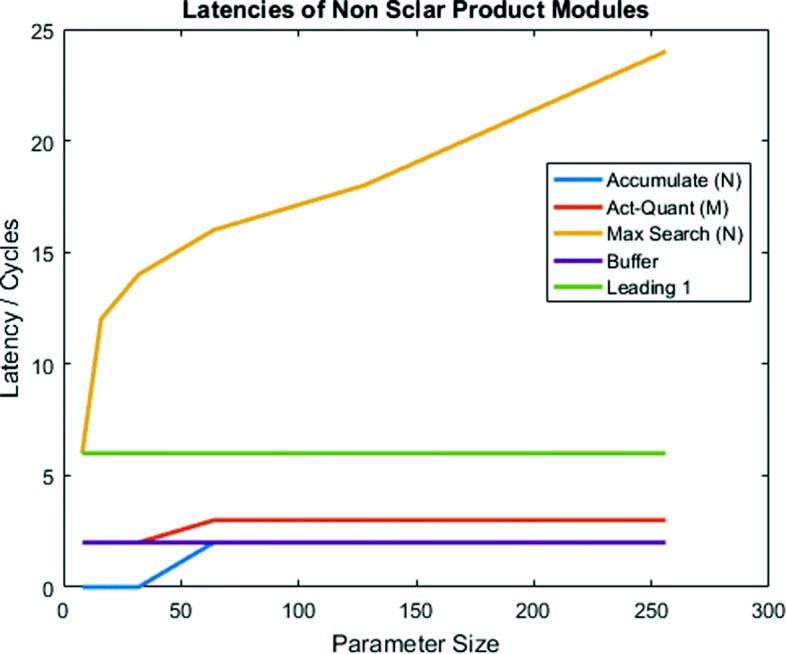
Latency of critical path modules based on their constraining parameter

Figure 8 illustrates the latency of modules in the Persistent Critical Path (except for Scalar Product accumulation). As was demonstrated with our system model previously, most modules have latency offsets based on pipeline depth and thus have nearly invariant latencies with respect to their associated parameter. With regard to Max Value Search, however, we get very large latencies despite it being a tree-based implementation. This is because of the resource overhead of a signed comparator as compared with a simple adder (≈2x more ALMs per comparator based on synthesis results).

**Fig. 9:**
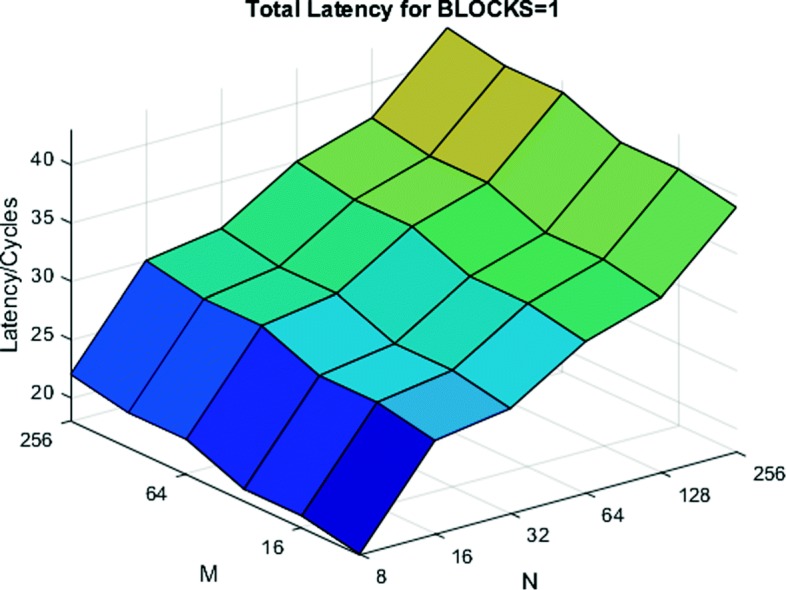
Total latency of the system for a single iteration. Having a larger value of M results in significantly lower latency than larger values of N

Figure 9 shows the total latency of our system for a single iteration of all modules. We observe that having larger values of M, instead of N, reduces latency by 20% on average.

### Resource usage

We have tested our designs on the Altera Arria-10AX115 FPGA. Table 3 gives the post-fit resource usage of the processor. We compile the design with *M* = 256 and *N* = 8. These values minimize overall inference latency based on the dimensions of benchmark models, Eq. 2, and latency results from Fig. 9. From the resource usage, we see that the design occupies less than half the chip. Therefore, based on Eq. 2, either a larger value of M can be used to further reduce latency, or a second (independent) inference processor can be included.

**Table 3:** FPGA implementation details

M	N	ALM	DSP	Frequency
256	8	57008/427200 (13%)	512/1518 (34%)	295 MHz

### Performance

**Fig. 10:**
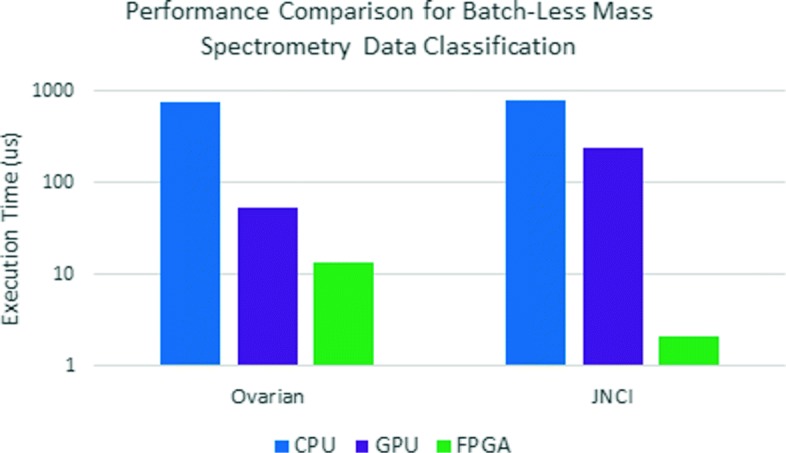
Performance Comparison between CPU, GPU, and FPGA. FPGA outperforms both the CPU and GPU with average speedups of 144x and 21x, respectively

We compare the performance of the FPGA implementation with an eight-core 2.6 GHz Intel Xeon E5-2650v2 CPU and an NVidia Tesla P100 GPU. Execution time for a single input test case is measured by performing inference for batch sizes shown in Table 1 and then taking the average. From the results (Fig. 10), we see that the FPGA outperforms the CPU and high-end GPU for both benchmarks, despite only using half the chip resources. FPGA execution time is 56x and 372x faster than CPU and 4x and 112x faster than GPU for the Ovarian and JNCI benchmarks, respectively.

## Discussion

The hurdle to performing real-time analysis on patient data is meeting timing constraints. This is difficult to do on traditional, fixed-logic platforms because of a large number of sources of overhead. Meeting timing constraints with fixed-logic platforms may continue to become less feasible. With technological advancements and greater integration of sensors into medical procedures, both the data throughput of individual sensors and their overall number can be expected to increase. Our results highlight the importance of application-specific optimizations for minimizing the latency of MLP inference and meeting significantly stricter performance requirements.

When constructing modules, several factors must be considered when determining parameter sizing, such as the impact on VCP and PCP. For PCP, when standard optimizations were applied using OpenCL-based architecture generation, the size and complexity of basic components, along with the nature of the overall computation, had a significant impact on the module latency. This is nearly invariant of the application itself and depends more on the design strategies for implementing individual modules. The VCP, on the other hand, is significantly more application-specific since it depends on the ratio of parameter values to nearly every layer size in the model. Thus, while there is an initial bias toward increasing the size of M over N, all latency contributions must be considered on a per-application basis to determine the optimal architecture.

An important observed trend in the performance results emphasizes the impact of batch-less execution on traditional accelerators. As the size of the model decreases, from Ovarian to JNCI, the FPGA execution time is also reduced since fewer computations are required for the smaller first hidden layer in JNCI. However, the GPU performance for the same transition shows a decrease. The reason is that GPUs depend heavily on batch processing of multiple test vectors in order to get good utilization of the thousands of computing cores. As a result, the lower number of test vectors for JNCI results in worse GPU performance despite there being fewer overall computations. Overall, we expect the CPU and GPU performance values to be even lower if batch-less test sets are run (as opposed to computing the average).

## Conclusion

We show the importance of application-specific optimizations in order to minimize latency and maximize resource utilization for MLP inference. By directly interfacing with and processing sensor data during procedures, FPGAs can perform real-time analysis and provide diagnostic feedback that can be critical to achieving higher percentages of successful patient outcomes. We propose a modular architecture that enables modifications to be easily performed based on the application model, design updates, and resource availability as the design is migrated to different FPGAs. We demonstrate that correct parameter sizing, based on the application, can reduce latency by 20% on average. Further, we show that in an application with tightly coupled data-path and latency constraints, having a large amount of computing resources can actually reduce performance. Moreover, since the FPGA does not require batch processing of inputs, it can operate with ultra-low latency in order to meet real-time constraints. Using mass-spectrometry benchmarks, our proposed FPGA design outperforms both CPU and GPU implementations with average speedups of 144x and 21x,respectively.

## Abbreviations

ALM: Adaptive logic module
ALU: Arithmetic logic unit
BCI: Brain-computer interface
COTS: Commercial-off-the-shelf
CADx: Computer-aided diagnosis
DNN: Deep neural network
DSP: Digital signal processor
FPGA: Field programmable gate arrays
GRU: Gated recurrent unit
GPIO: General purpose input/output
GPU: Graphics processing unit
HBM: High bandwidth memory
LSTM: Long short-term memory
ML: Machine learning
MVM: Matrix vector multiplication
MSB: Most significant bit
MGTs: Multi-gigabit transceivers
MLP: Multi-layer perceptron
PCP: Persistent critical path
RF: Rotation forest
SVM-RFE: Support vector machine recursive feature elimination
SELDI: Surface-enhanced laser desorption and ionization
VCP: Variable critical path
